# A combined approach with gene-wise normalization improves the analysis of RNA-seq data in human breast cancer subtypes

**DOI:** 10.1371/journal.pone.0201813

**Published:** 2018-08-08

**Authors:** Xiaohong Li, Eric C. Rouchka, Guy N. Brock, Jun Yan, Timothy E. O’Toole, David A. Tieri, Nigel G. F. Cooper

**Affiliations:** 1 Department of Anatomical Sciences and Neurobiology, University of Louisville, Louisville, KY, United States of America; 2 Department of Computer Engineering and Computer Science, University of Louisville, Louisville, KY, United States of America; 3 Department of Biomedical Informatics, Ohio State University, Columbus, OH, United States of America; 4 Department of Medicine, James Graham Brown Cancer Center, University of Louisville, Louisville, KY, United States of America; 5 Department of Cardiology, University of Louisville, Louisville, KY, United States of America; University of South Alabama Mitchell Cancer Institute, UNITED STATES

## Abstract

Breast cancer (BC) is increasing in incidence and resistance to treatment worldwide. The challenges in limited therapeutic options and poor survival outcomes in BC subtypes persist because of its molecular heterogeneity and resistance to standard endocrine therapy. Recently, high throughput RNA sequencing (RNA-seq) has been used to identify biomarkers of disease progression and signaling pathways that could be amenable to specific therapies according to the BC subtype. However, there is no single generally accepted pipeline for the analysis of RNA-seq data in biomarker discovery due, in part, to the needs of simultaneously satisfying constraints of sensitivity and specificity. We proposed a combined approach using gene-wise normalization, UQ-pgQ2, followed by a Wald test from *DESeq2*. Our approach improved the analysis based on within-group comparisons in terms of the specificity when applied to publicly available RNA-seq BC datasets. In terms of identifying differentially expressed genes (DEGs), we combined an optimized log_2_ fold change cutoff with a nominal false discovery rate of 0.05 to further minimize false positives. Using this method in the analysis of two GEO BC datasets, we identified 797 DEGs uniquely expressed in triple negative BC (TNBC) and significantly associated with T cell and immune-related signaling, contributing to the immunotherapeutic efficacy in TNBC patients. In contrast, we identified 1403 DEGs uniquely expressed in estrogen positive and HER2 negative BC (ER^+^HER2^-^BC) and significantly associated with eicosanoid, notching and FAK signaling while a common set of genes was associated with cellular growth and proliferation. Thus, our approach to control for false positives identified two distinct gene expression profiles associated with these two subtypes of BC which are distinguishable by their molecular and functional attributes.

## Introduction

Breast cancer (BC) is the most commonly diagnosed cancer in women throughout the world [1–3], accounting for 23% of all female cancers [4–6]. BC is a growing health problem worldwide, increasing both in incidence [3] and resistance to treatment. Although significant progress has been made in the clinical treatment of BC, challenges persist because of its molecular heterogeneity, resistance to standard endocrine therapy and the risk of late recurrence. These challenges are driving intense research efforts to identify new biomarkers of disease progression and signaling pathways to aid in diagnosis or treatment.

Since BC exhibits heterogeneity, the identification of molecular markers, gene expression profiles and patterns of genomic alteration used as analytic tools is essentially required for predicting clinical outcomes and selecting appropriate therapies [7]. In particular, the presence of estrogen and progesterone receptors (ER and PR), and the human epidermal growth factor receptor 2 (*HER2*) have become standard biomarkers for defining BC subtypes which can be targeted by hormone modulation therapy. Approximately 75% of all BC are ER^+^ and of these, only half respond to anti-estrogen therapy [8,9]. ER^+^ patients ultimately comprise the majority of deaths attributable to BC. Therefore, finding new putative targets for chemotherapy is an urgent need [10–12]. Studies of ER^+^BC have demonstrated that ER signaling engages in complex cross-talk encompassing multiple signaling pathways with both genomic and non-genomic involvement [8,13]. ER^+^BC is associated with enhancing cellular proliferation either by increasing cell division and/or decreasing apoptosis [13,14]. On the other hand, tumors lacking ER and PR as well as *HER2* (triple negative breast cancers, TNBC) are not amenable to these targeted therapies. Studies report that TNBC is more sensitive to chemotherapy than hormone positive BC [7,15–17]. However, TNBC is associated with poorer survival than non-TNBC due to frequent relapse, and only about 31% of patients are completely responsive to chemotherapy [15,16,18].Therefore, a better understanding of the cellular and molecular pathways underlying BC initiation and progression remains necessary for improving therapeutic options and clinical outcomes.

High throughput RNA sequencing (RNA-seq) has been increasingly used in clinical studies for defining changes in gene expression [19,20]. Indeed, RNA-seq-based gene expression profiling for the identification of global gene-expression patterns is commonly used to integrate the multiple molecular events and mechanisms associated with the development of cancer [5,21]. The mechanisms of oncogenesis involve the disruption of diverse biological functions and cellular pathways including cell cycle, proliferation, survival and apoptosis [5]. However, the development of a standard approach for the analysis of DEGs has been problematic due to the multiple analytical steps required in typical RNA-seq workflow. Of these steps, normalization is critical for appropriately comparing different sets of samples [22].

Studies comparing normalization methods and statistical testing packages have shown that normalization methods have a strong impact on the outcomes of analysis [22–28]. Using simulated data, it was found that normalization methods such as TC (Total Counts), UQ (Upper Quantile), Med (Median), FQ (Full Quantile) and RPKM normalization methods failed to control the false positive rate for genes with high read counts[22,23]. In contrast, the DESeq (*DESeq* and *DESeq2*) [29,30] and TMM (*edgeR*) [31] methods performed better overall than other methods in terms of detection power and control of false positives in data at a specified false discovery rate (FDR) [23,24]. However, these studies reported that an observed type I error rate was higher than the nominal FDR, leading to an inflated type I error rate. More recently, gene-wise normalization methods following per sample globally scaled normalization (UQ-pgQ2 and Med-pgQ2) were proposed [28]. A comparison of these methods with *DESeq* normalization from *DESeq2* and TMM normalization from *edgeR* using the benchmark Microarray Quality Control Project datasets (MAQC2) [22] reported that Med-pgQ2 or UQ-pgQ2 performed slightly better for genes with high read counts by improving the specificity for skewed RNA-seq data given a FDR of 0.05. However, these gene-wise normalization methods showed a slightly higher FP (false positive) rate for genes with a mean read counts below the 25th percentile compared to *DESeq2* and *edgeR* [28].

In this study, a new approach was used to perform within-group comparison analysis using publicly available RNA-seq datasets including GEO ER^+^HER2^-^BC, TNBC [32] and The Cancer Genome Atlas (TCGA) BRCA datasets (https://cancergenome.nih.gov/publications). We observed that the normalization with the DESeq and UQ-pgQ2 methods followed by a Wald test from *DESeq2* performed better than TMM from *edgeR* based on the type I error rate or specificity. We found *edgeR* identified a higher number of FP genes using RNA-seq datasets. To further minimize the FP rate and maximize the true positive DEGs, we integrated the results from these two methods by robustly selecting an optimal |logFC| cutoff at which the observed FP rate from the within group comparison is minimized and a reasonable number of true DEGs identified. With this combined approach, we performed the analysis of DEGs on the GEO TNBC and ER^+^HER^-^BC data by comparing BC versus normal control. Three sets of DEGs were identified, including two DEG sets uniquely expressed in either of the TNBC or ER^+^HER^-^BC groups and one common DEG set identified in both BC subtypes. These DEGs were further analyzed for biological functions and pathways with the aid of the Ingenuity Pathway Analysis software (IPA). These gene expression profiles are distinguishable by their molecular and functional attributes associated with distinct functions and signaling pathways.

## Materials and methods

### Normalization methods

Three normalization methods (DESeq, TMM and UQ-pgQ2), and two software packages for determining differential expression (*DESeq2* and *edgeR)* were used in our study [28–31]. DESeq and TMM normalization methods were implemented using the *DESeq2* and *edgeR* packages, respectively (Table 1). UQ-pgQ2 normalization was implemented using R.

**Table 1:** Summary of normalization methods and software packages used.

Normalization method	Description of normalization	Distribution	Statistical test	Software packages
UQ-pgQ2	Per sample scaled by upper quantile and per gene by medium across samples	NB	Wald test	*DESeq2* (v1.6.3)
DESeq	Per sample scaled by medium of ratio	NB	Wald test	*DESeq2* (v1.6.3)
TMM	Per sample by Trimmed Mean M values	NB	Exact test	*edgeR* (v3.8.6)

NB: a negative binomial distribution.

### Data sources

The publicly available RNA-seq datasets contain forty-two TNBC primary tumors; twenty-one uninvolved breast tissue samples adjacent to TNBC primary tumors (ctr1); forty-two Estrogen Receptor positive (ER^+^) and HER2 negative (HER2^-^) breast cancer (ER^+^HER2^-^BC) primary tumors and 30 uninvolved breast tissue samples adjacent to ER^+^HER2^-^BC primary tumors (ctr2). The RNA-seq raw data files were downloaded from NCBI GEO and SRA (series ID GSE58135) [32].

The third paired breast cancer data with raw gene read counts contains 117 primary tumors and 112 uninvolved breast tissue samples adjacent to the primary tumors (ctr) which were downloaded from The Cancer Genome Atlas website: http://portal.gdc.cancer.gov/projects/TCGA-BRCA. The 117 tumor samples paired with 112 normal controls were extracted from 1098 TCGA-BRCA cases. To confirm our findings, an additional 122 TNBC samples were extracted from 1098 TCGA-BRCA cases and used for the within-group analysis (https://cancergenome.nih.gov/publications).

### Sequence mapping and extraction of gene read counts

The raw SRA sequencing files downloaded from GEO were first converted to .fastq files and subsequently mapped to the human hg19 reference genome using STAR (v2.5.3a) [33]. The mapped counts for 57,778 genes per sample were then extracted using HTSeq-scripts-count (version 2.7). After filtering the genes with zero counts across all the samples with four groups, 35,203 genes per sample were left for downstream analysis.

The downloaded TCGA-BRCA data containing 56,963 genes with raw reads was preprocessed by filtering out genes with zero read counts across 117 tumors and 112 normal samples. Thus, a total of 35,113 genes were used for within and between group comparisons for identifying the best method.

### Software packages used for normalizing and testing DEGs

The normalization methods, software packages, and test statistics used for analysis are summarized in Table 1. Briefly, *edgeR* (v3.8.6) [31] implements TMM normalization and has been widely used for DEG analysis for RNA-seq data. *DESeq2* [30], a successor to *DESeq* [29], implements DESeq normalization and a Wald statistical test for detection of DEGs. Following UQ-pgQ2 normalization [28], *DESeq2* was used for identifying DEGs.

### Normalization method for downstream analysis

In order to control for false positives, DEGs analysis of six within-group comparisons was performed: 21 TNBC vs. 21 TNBC, and 11 ctr1 vs. 10 ctr1 (control for TNBC); 21 ER+HER-BC vs. 21 ER^+^HER2^-^BC, and 15 ctr2 vs. 15 ctr2 (control for ER^+^HER2^-^BC); 59 TCGA-BRCA vs. 58 TCGA-BRCA, and 56 ctr vs. 56 ctr (paired control for TCGA-BRCA). Since the samples originate from the same condition (within-group), it is expected there should be relatively few, if any, true DEGs, and thus any detected DEGs can be treated as FP genes. All the samples in each condition were equally and randomly divided into two groups. For each group, we repeated the procedure 10 times by randomly sampling without replacement using an R script to account for individual sample variances. We then determined the optimal |logFC| cutoff for each normalization method to minimize FP genes with and FDR ≤ 0.05. This cutoff was determined based on an observed false positive error rate (FPR) ≤ 0.05%.

### Identification of true DEGs for the comparisons of BC versus control

We performed DEGs analysis for two comparisons: TNBC versus control and ER^+^HER2^-^BC versus control using the UQ-pgQ2 and *DESeq2* methods. DEGs were determined by *DESeq2* using the optimal |log FC| cutoff that minimizes the FPR (as determined by the within-group comparisons from the previous section). For each comparison, we assumed DEGs identified in common using both methods were true positive (TP) DEGs. In addition, genes above the optimal |logFC| cutoff value identified either by *DESeq2* or UQ-pgQ2 were also considered as TP DEGs. The TP DEGs identified from TNBC and ER^+^HER2^-^BC were further analyzed for discovery of the common or unique genes in two BC subtypes (TNBC and ER^+^HER2^-^BC).

### Biological function and pathway analysis

We used IPA to identify the distinct biological functions and canonical signaling pathways giving the two sets of gene expression profiles uniquely expressed in TNBC and ER^+^HER2^-^BC patients (QIAGEN, version 3355999, USA) as a manner of validating the functions of genes determined to be differentially expressed.

## Results

### Comparison of normalization methods

DEGs identified from the within-group comparisons of the four BC datasets using UQ-pgQ2, *DESeq2* and *edgeR* are listed in Table 2, S1 and S2 Tables. The results with a varying |logFC| cutoff show that UQ-pgQ2 is more conservative than the other methods, resulting in lower FP rates. For the within-group comparisons (21 TNBC vs. 21 TNBC; 11 ctrl vs. 10 ctrl; 21 ER^+^HBR^-^BC vs. 21 ER^+^HBR^-^BC; 15 ctrl2 vs. 15 ctrl2), UQ-pgQ2 consistently has low FP rates, with fewer than 10 FP DEGs determined at |Log(FC)| cutoff of 1.5, and no FP DEGs determined for higher cutoffs (Table 2). *DESeq2* performs at a high level as well, with a FPR ranging from 0 to 0.12%. *edgeR* yields higher numbers of FP DEGs, with an FPR up to 1.5%. Given the results listed in Table 2 for both UQ-pgQ2 and *DESeq2*, a |logFC| of 2 was chosen as an optimum cutoff value for the downstream analysis of DEGs since it minimizes the FPR within an acceptable threshold. Increasing the |logFC| cutoff to 2.5 nearly eliminates the FPR for both UQ-pgQ2 and *DESeq2*, while *edgeR* maintains an FPR > 1%.

**Table 2:** DEG analysis performed via within-group and between-group comparisons from three methods. The DEGs from between–group comparisons in bold are determined given a FDR ≤ 0.05.

|Log(FC)|	Comparison groups	UQ-pgQ2	*DESeq2*	*edgeR*
≥1.5	21TNBC vs. 21 TNBC	4±4	43±34	527±125
	11ctr1 vs. 10 ctr1	1±2	0	6±14
	21 ER+HER2-BC vs. 21 ER+HER2-BC	1±1	6±3	292±67
	15 ctr2 vs. 15 ctr2	1±3	14±16	771±184
	**42 TNBC vs. 21 ctr1**	**7,474**	**8,969**	**9,585**
	**42 ER+HER2-BC vs. 30 ctr2**	**4,999**	**6,308**	**7,448**
≥2	21TNBC vs. 21 TNBC	0	10±9	455±97
	11ctr1 vs. 10 ctr1	0	0	6±12
	21 ER+HER2-BC vs. 21 ER+HER2-BC	0	1±1	259±54
	15 ctr2 vs. 15 ctr2	0	5±6	686±137
	**42 TNBC vs. 21 ctr1**	**3,706**	**5,201**	**5,854**
	**42 ER+HER2-BC vs. 30 ctr2**	**2,169**	**3,176**	**4,161**
≥2.5	21TNBC vs. 21 TNBC	0	1±1	372±74
	11ctr1 vs. 10 ctr1	0	0	5±10
	21 ER+HER2-BC vs. 21 ER+HER2-BC	0	0	216±39
	15 ctr2 vs. 15 ctr2	0	1±2	590±94
	**42 TNBC vs. 21 ctr1**	**1,701**	**2,888**	**3,586**
	**42 ER+HER2-BC vs. 30 ctr2**	**869**	**1,499**	**2,326**
≥3	21TNBC vs. 21 TNBC	0	0	296±54
	11ctr1 vs. 10 ctr1	0	0	4±7
	21 ER+HER2-BC vs. 21 ER+HER2-BC	0	0	175±33
	15 ctr2 vs. 15 ctr2	0	0	502±65
	**42 TNBC vs. 21 ctr1**	**767**	**1815**	**2,290**
	**42 ER+HER2-BC vs. 30 ctr2**	**323**	**689**	**1,356**

We also observed a high FPR from *edgeR* and a low FPR from UQ-pgQ2 for DEG analysis of 117 paired TCGA-BRCA and 112 control samples using the within group approach (S1 Table), in this case, giving a |logFC| cutoff set at 1, 1.5, 2 or 3. For the 59 TCGA-BRCA versus 58 TCGA-BRCA comparison, the number of FP genes with a |logFC| ≤1 cutoff using UQ.pgQ2, *DESeq2* and *edgeR* is 70±97, 120±160 and 2019±789, respectively, with an observed FPR of 0.20%, 0.34% and 5.75% for the 35,113 genes measured. The number of FP genes in the normal control comparison (56 control vs. 56 control) from UQ-pgQ2, *DESeq2* and *edgeR* is 2±3.3, 4±5 and 513±47, respectively, with a FPR of 0.006%, 0.011% and 1.45%. This indicates the importance of using multiple cutoffs, since the number of FP DEGs increases significantly when a |logFC| under 1.5 is used. Given a |logFC| of 2, the number of FP genes in TCGA-BRCA for UQ.pgQ2, *DESeq2* and *edgeR* is 8 ±10, 18±23 and 1,050±514, with an observed FPR of 0.02%, 0.05% and 3.00% respectively, while the number of FP genes in the normal control comparison is 0, 0 and 308±21, with a FPR of 0.003%, 0.006% and 1.13%, respectively.

For the TCGA datasets, a |logFC| cutoff of 2 was chosen as an optimum FC cutoff value for identification of DEGs given a nominal FDR <0.05 and an observed error rate below or close to 0.05% for UQ.pgQ2 and *DESeq2* with 2,148 and 2,208 DEGs identified, respectively.

Since the TCGA data yields higher FPR for all approaches comparing with the BC datasets downloaded from GEO, indicating an increased variance for these samples, it may in part be due to the lack of separation in BC subtypes. To address this concern, we further extracted 122 TNBC samples from TCGA-BRCA based on clinical information. A within-group comparison (61 TCGA-TNBC and 61 TCGA-TNBC) was performed. The results in S2 Table consistently showed that UQ.pgQ2 and *DESeq2* outperformed *edgeR* in terms of the control of FPR.

In summary, an approach via the within-group analysis to identify FP genes can help to achieve several goals. First, among three methods, we observe that UQ-pgQ2 and *DESeq2* outperformed *edgeR* for controlling type I error rate while UQ-pgQ2 was slightly better than *DESeq2* overall. This finding is consistent with the report from our previous study [28]. Second, the results (Table 2, S1 and S2 Tables) suggest that UQ-pgQ2 is more conservative than *DESeq2* in most datasets while *edgeR* performs comparatively worst for all datasets. This observation is consistent with our previous findings while comparing normalization methods for the analysis of DEGs within RNA-seq data [28]. Finally, the results (Table 3) helped to choose an optimal |logFC| by taking into consideration of a FPR with a good detection power for a reasonable number of DEGs.

**Table 3:** Determining an optimal |logFC|** by observed FPR. An observed FPR based on all of 35203 genes is computed given a |logFC| cutoff in parenthesis.

	Normalization	DEGs	FPR* (|logFC|)	|logFC|** given FPR≈0
TNBC	UQpgQ2	3,706	≈0 (≥2)	≥2
	DESeq2	5,201	≈0.03% (≥2)	≥2.5
	Common DEGs	3,610	-	-
ER+HER2-BC	UQpgQ2	4,999	≈0.003% (≥1.5)	≥2
	DESeq2	6,308	≈0.04% (≥1.5)	≥2
	Common DEGs	4,776	-	-

FPR*: false positive rate

|logFC|**: maximum cutoff value.

### 2. DEGs identified between-group comparisons in human TNBC and ER^+^HER2^-^BC from three methods

Gene expression profiles in two comparisons (42 human TNBC versus 21 controls (ctr1), and 42 human ER^+^HER2^-^BC versus 30 controls (ctr2)) were analyzed using three methods (UQ-pgQ2, *DESeq2* and *edgeR*).

DEGs are identified giving a nominal FDR ≤0.05 and an optimal |logFC| cutoff value (Tables 2 and 3). The results in bold (Table 2) show that *edgeR* has a higher detection power while having a tradeoff of a higher FPR given the same |logFC| cutoff according to the within-group analysis. Although *DESeq2* performs better in terms of FPR when compared to *edgeR*, the actual type I error in *DESeq2* is higher than the nominal FDR, particularly in high read counts of genes based on previous studies using simulated data. With the aid of *DESeq2*, UQ-pgQ2 has a much lower FPR while having a tradeoff of a fewer number of DEGs detected. In order to maximize the detection power and minimize the type I error, we utilized UQ-pgQ2 and *DESeq2* to identify the DEGs given a nominal FDR of 0.05 and an optimal |logFC| cutoff (Table 3).

The results in Table 3 show that using UQ-pgQ2 method, 3,706 DEGs in TNBC and 4,999 DEGs in ER^+^HER2^-^BC are detected given an optimal |logFC| cutoff of 1.5 and 2 with an observed FPR below 0.002%. Similarly, using *DESeq2*, 5,201 DEGs in TNBC and 6,308 DEGs in ER^+^HER2^-^BC are detected given the same |logFC| cutoff as UQ-pgQ2 with an observed FPR below 0.03%.

### 3. DEGs identified in human TNBC and ER^+^HER2^-^BC from UQ-pgQ2 and *DESeq2* based on 17,584 protein coding genes

Gene expression profiling is commonly used to identify disease biomarkers and biological functions. In RNA-seq data, we have noted that the identified DEGs contain a mixture of mRNA, miRNA, rRNAs and other non-coding RNAs that are present in the total RNA per sample. These noncoding RNAs, especially about 10% of high abundant rRNAs with high read counts are not completely eliminated and remain in each RNA-seq sample while using the ribosomal depletion method in the library preparations. In this study, we focused on the 17,584 protein coding genes out of the 35,203 total genes. The results in Table 4 show that using UQ-pgQ2 method, the number of DEGs detected for TNBC at a |logFC|≤2 and ER^+^HER2^-^BC at a |logFC|≤ 1.5 is 1,584 and 2,303, respectively; using *DESeq2* method with the same cutoff values, the number of DEGs detected for TNBC and ER^+^HER2^-^BC is 1,913 and 2,649, respectively. The number of DEGs common in both analytical methods for TNBC and ER^+^HER2^-^BC is 1,546 and 2,212, respectively. In addition, Table 4 also displays the number of up and down-regulated DEGs per comparison.

**Table 4:** DEGs identified using *DESeq2* and UQ-pgQ2. The DEGs from 17,584 protein coding genes are determined given a nominal FDR ≤0.05 and an optimal |logFC| cutoff in Table 3.

Data	Normalization	DEGs	Up	Down
TNBC	UQ.pgQ2	1,584	949	635
	DESeq2	1,913	1099	814
	Common DEGs	1,546	915	631
ER^+^HER2^-^BC	UQ.pgQ2	2,303	1,161	1,142
	DESeq2	2,649	1,195	1,454
	Common DEGs	2,212	1,074	1,138

### 4. Robust identification of the true DEGs (protein coding genes) from UQ.pgQ2 and *DESeq2*

Based on the DEG analysis of the 17,584 protein coding genes, we noted that the number of DEGs identified by the UQ-pgQ2 and *DESeq2* methods varied for the two comparisons (42 TNBC versus 21 control and 42 ER^+^HER2^-^BC versus 30 control). The previous studies observed that *DESeq2* and *edgeR* were less conserved for the high read count genes using MAQC2 data [28]. Therefore, in order to minimize the number of false positives and maximize the true DEGs, we used a combined approach to identify the true DEGs. The results were listed in Table 3. For the TNBC comparison, we first identified the common DEGs between UQ-pgQ2 and *DESeq2* resulting in 1,546 DEGs. Similarly, for the ER^+^HER2^-^BC comparison, 2,212 DEGs were identified in common. We assumed the DEGs that were not in common, but identified by either *DESeg2* or UQ-pgQ2 with an observed FPR close to zero given a Max |logFC| cutoff (Table 3), were also considered as true DEGs. With this approach, in the TNBC comparison, 109 DEGs from *DESeq2* at a |logFC| ≥2.5, and 38 DEGs from UQ-pgQ2 at a |logFC| ≥2, were considered as true DEGs, and adding them to the common DEGs set resulted in 1,693 true DEGs. For the ER^+^HER2^-^BC comparison, with a |logFC| ≥2, 84 DEGs from *DESeq2* and 3 DEGs from UQ-pgQ2 were considered as the true DEGs, and adding them to the common DEG set resulted in 2,299 true DEGs. The results were listed in Table 5. A Venn diagram (S1 Fig) illustrates the common and unique genes between 1693 DEGs in TNBC and 2299 DEGs in ER^+^HER2^-^BC.

**Table 5:** An approach to select DEGs (protein coding genes) identified by UQ-pgQ2* and *DESeq2*.

	Common	UQ-pgQ2*	DESeq2	Total
TNBC	1,546	38 (|logFC|≥2)	109 (|logFC|≥2.5)	1,693
ER+HER2-BC	2,212	3 (|logFC|≥2)	84 (|logFC|≥2)	2,299

UQ-pgQ2*: UQ-pgQ2 normalization and Wald test from *DESeq2*.

**Fig 1:**
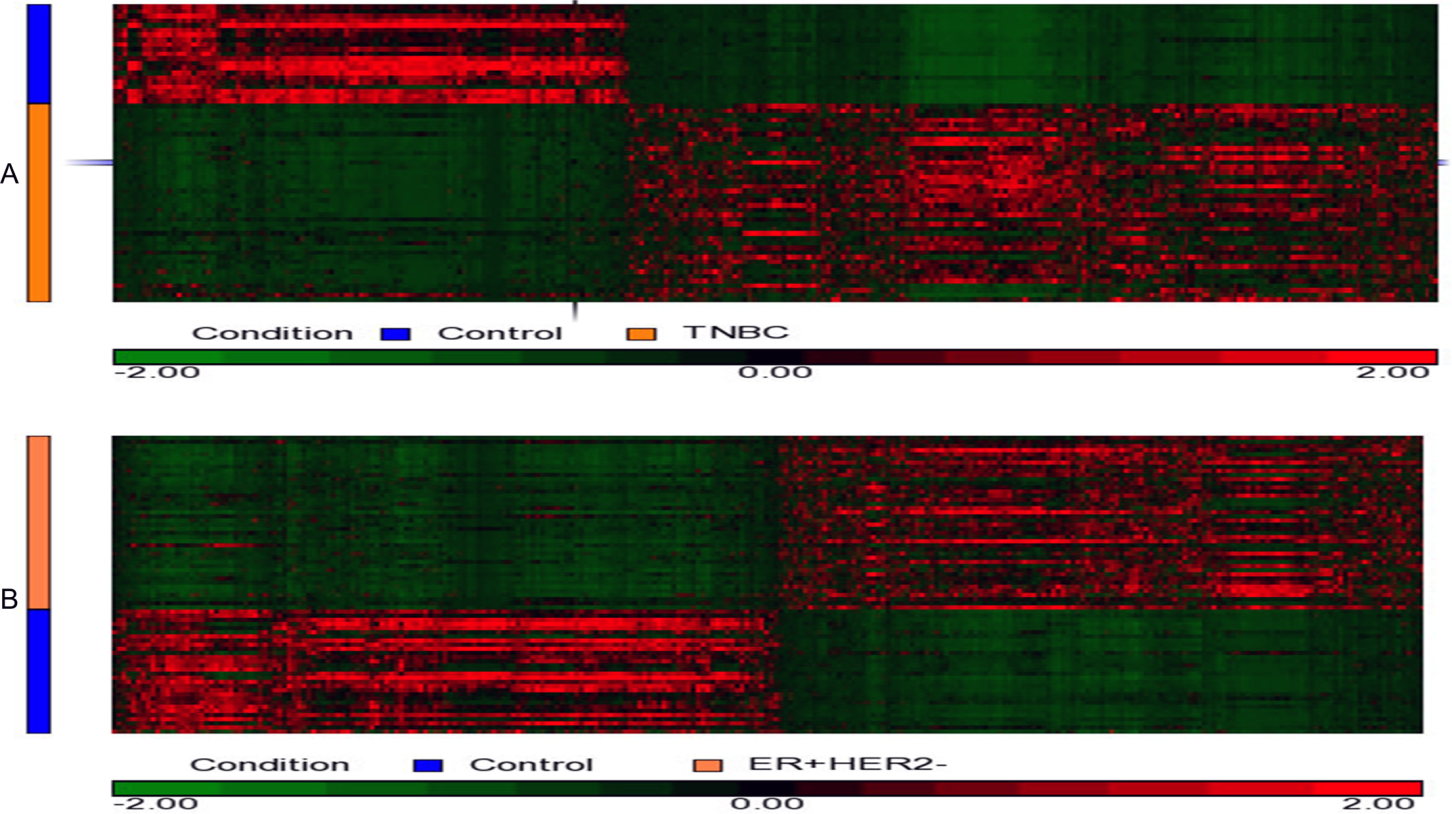
Hierarchical clustering heatmaps of BC based on the DESeq-normalized gene expression levels. The genes with similar expression patterns are clustered together. The up-regulated genes are in red and the down-regulated genes are in green. (A) A heatmap based on gene expression levels of 1,693 DEGs uniquely identified in TNBC data. (B) A heatmap based on gene expression of 2,299 DEGs uniquely identified in ER^+^HER2^-^BC data.

The heatmaps (Fig 1) based on the *DESeq2*-normalized gene expression levels were constructed using hierarchical clustering from Partek software (Partek Genomics Suite 6.6). In this figure, the up-regulated genes in red and down-regulated genes in green were conventionally chosen. Fig 1A illustrates the gene expression level of the 1,693 DEGs for the 42 TNBC versus 21 control samples. Fig 1B illustrates the gene expression level of the 2,299 DEGs for the 42 ER^+^HER2^-^BC versus 30 control samples.

Finally, the number of common and unique DEGs between the 1,693 DEGs in TNBC and the 2,299 DEGs in ER^+^HER2^-^BC was examined (Table 5 and S1 Fig). The 896 DEGs common in both include the top 10 up-regulated genes: *IBSP*, *FRAME*, *COL10A1*, *HMX2*, *HIST1H31*, *ASPM*, *KIF14*, *MMP11* and *CENPF*; and the top 10 down-regulated genes: *MYOC*, *SLC22A12*, *LEP*, *PLIN4*, *PLIN1*, *GLYAT*, *GPD1*, *ADIPOQ*, *HBB* and *CIDEC* (S3 Table). There are 797 DEGs uniquely identified in TNBC including the top 10 up-regulated genes: *MMP13*, *VAX1*, *PSAPL1*, *LHX2*, *HORMAD1*, *CCKBR*, *KIF1A*, *COL22A1*, *SIX3*, *CXCL13* and *POU4F1*; and the top 10 down-regulated genes: *CES1*, *HSD17B13*, *PCK1*, *RBP4*, *AGTR1*, *CLSTN2*, *MASP1*, *ACSM5*, *PTGER3*, *SLC5A7* (S4 Table). There are 1403 DEGs uniquely identified in ER^+^HER2^-^BC including the top 10 up-regulated genes: *CBLN2*, *SLC30A8*, *VSTM2A*, *GRM4*, *FOX11*, *RIMS4*, *SERPINA12*, *SYT1*, *IGFL1* and *EEF1A2*; and the top 10 down-regulated genes: *FGFBP2*, *SPHKAP*, *XDH*, *SLC22A3*, *SLCO1B7*, *KCNB1*, *SERTM1*, *AKR1B15*, *ACSL1* and *BMP3* (S5 Table). The DEGs were further used for the analysis of the cancer-related biological functions and pathways with the aid of IPA (http://www.ingenuity.com/).

### 5. Identification of biomarker genes based on the presence or absence ER, PR and HER2 to partially validate the DEGs analysis

We identified biomarker genes based upon the presence or absence of the molecular receptors (Table 6). For the TNBC comparison, we found that ER (*ESR1* and *ESR2*), PR (*PGR*) and HER2 (*EGFR*) were significantly down-regulated using both UQ.pgQ2 and *DESeq2* methods as expected. For the ER^+^HER2^-^BC comparison, we found that ER1 (*ESR1*) was significantly up-regulated with a FC greater than 1.8 and ER2 (*ESR2*) was significantly down-regulated. PR (*PGR*) expression level in ER^+^HER2^-^BC was not significantly different from the control groups. However, HER2 (EGFR) was significantly down-regulated using both methods as expected. Taken together, the expected results via the molecular markers can partially validate the true DEGs using an integrated approach.

**Table 6:** Biomarkers identified for TNBC and ER^+^HER2^-^BC.

Comparison	Symbol	LogFC		FDR
		UQ.pgQ2	DESeq2	
TNBC	*ERS1*	-2.95	-3.25	≤0.001
	*ERS2*	-0.94	-1.01	≤0.002
	*PGR*	-3.12	-3.56	≤0.001
	*EGFR (HER2)*	-1.51	-1.65	≤0.01
ER+HER2-BC	*ERS1*	0.92	0.84	≤0.005
	*ERS2*	-1.84	-2.01	≤0.001
	*PGR*	0.23	-0.36	≥0.59
	*EGFR (HER2)*	-3.23	-3.43	≤0.001

### 6. Top cancer-related biological functions and networks identified via IPA

**Fig 2:**
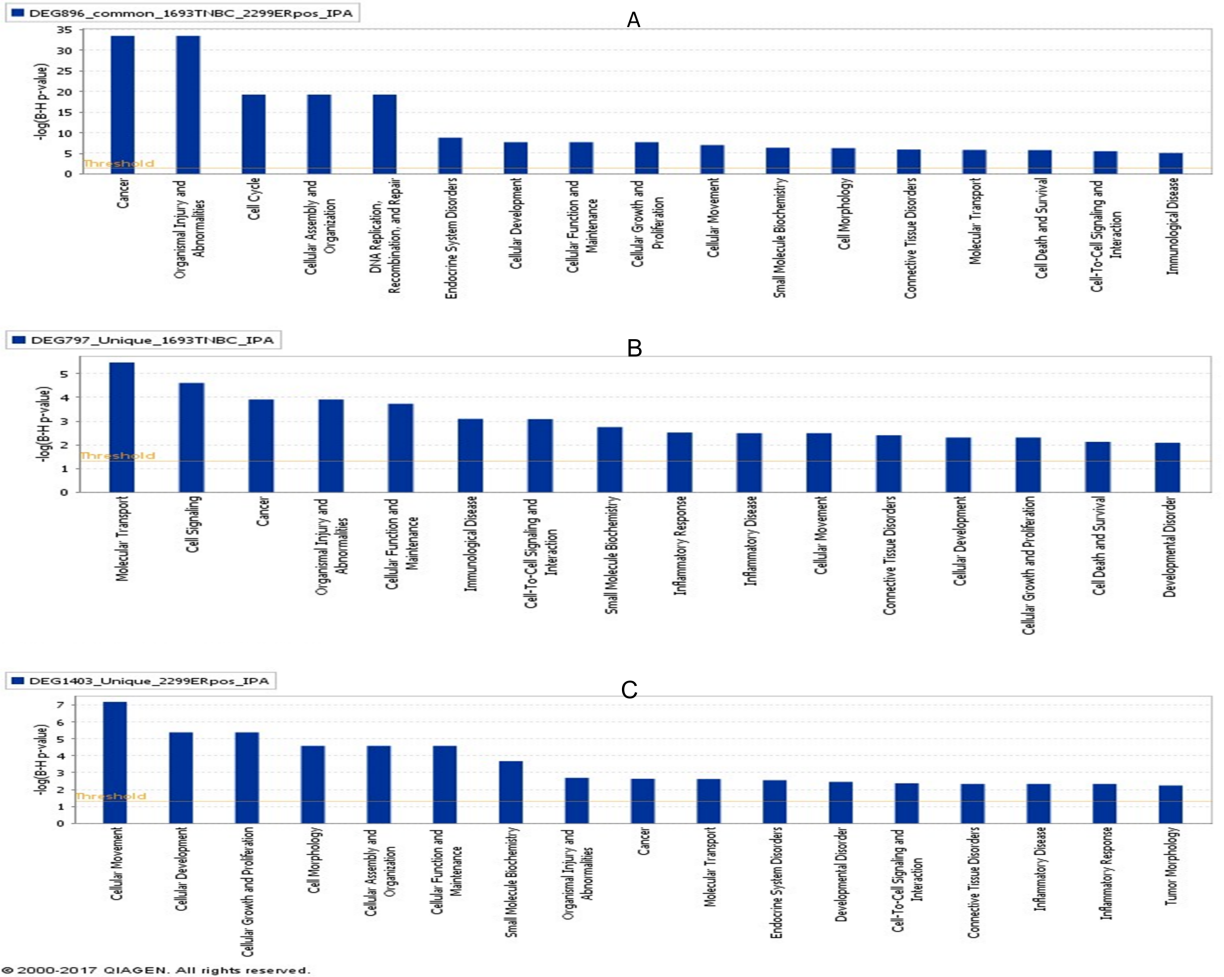
Biological functions of DEGs for BC subtypes identified by IPA. (A) Illustrated are the biological functions based on 896 DEGs commonly identified in TNBC and ER^+^HER2^-^BC. **(**B) Illustrated are the biological functions based on 797 DEGs uniquely identified in TNBC subtype. (C) Illustrated are the biological functions based on 1403 DEGs uniquely identified in ER+HER2-BC subtype.

**Fig 3:**
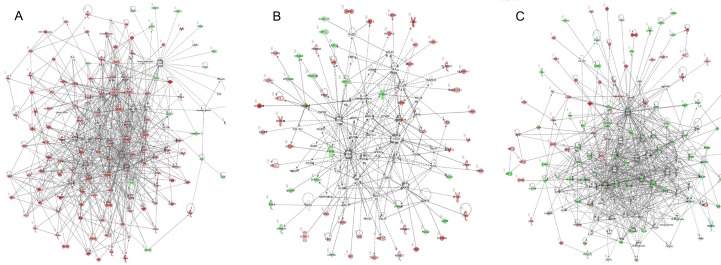
Top networks of DEGs identified by IPA. The networks are defined as Cancer, and Organismal Injury and Abnormalities by IPA. The up-regulated and down-regulated genes are in red and green, respectively. (A) The top network is based on 797 DEGs in TNBC. (B) The top network is based on 1403 DEGs in ER+HER2-BC.

IPA (http://www.ingenuity.com/) is a widely used tool for the partial validation, but mainly used in identification of diseases and biological functions. The three sets of common and unique DEGs (S1 Fig) were loaded to IPA and the results were obtained (Table 7 and Figs 2 and 3). We particularly focused on the cancer or immuno-related biological functions (Fig 2). Fig 2A illustrates the top diseases and biological functions significantly identified by the common set of DEGs. These are categorized as Cancer, Organismal Injury and Abnormalities, and Cell Cycle. Fig 2B highlights the top biological functions from the set of DEGs uniquely expressed in TNBC. These functions include Tissue Morphology, Cell Signaling, Immune Cell Trafficking and Inflammatory Response, Humoral Immune Response, Cell-mediated Immune Response, Cellular Movement and Development, Cellular Growth and Proliferation, Organismal Development and Morphology, and Cell Death and Survival. T cell-mediated immune response has been linked to the efficacy of immunotherapy in TNBC. In contrast, inflammatory response has been shown to promote tumor development and metastasis. Fig 2C highlights the top biological functions uniquely expressed in ER^+^HER2^-^BC that are associated with Cellular Movement and Development, Cellular Growth and Proliferation, Tissue and Organismal Development, T-cell Signaling, Immune Cell Trafficking and Inflammatory Response. We noted that the functions of Cellular or Tissue Movement, and Cellar Growth and Proliferations identified from the unique set of DEGs in ER^+^HER2^-^BC were much more significant than the functions categorized in Inflammatory Response and Immune Response. This observation suggests these functions may play a dominant role during ER^+^HER2^-^BC cell development and growth. In contrast, the functions categorized as Cell Signaling, Immune Cell Trafficking and Inflammatory Response, Humoral Immune Response etc. identified from the unique set of DEGs in TNBC were much more significant than the functions categorized as Cellar Growth and Proliferations, and Cell Death and Survival. This observation suggests Inflammatory Response or Cell-mediated Immune Response may play a dominant role for helping metastasis, which may be as a potential mechanism to explain why the TNBC patients has a poor survival rate. In addition, immune cell trafficking may play a critical role in TNBC immunotherapy. Indeed a recent clinical study revealed that immune checkpoint inhibitor therapy in TNBC has achieved about 19% of the overall response rate with durable clinical responses [34].

**Table 7:** The DEGs are associated with cancer biology identified by IPA.

	DEGs	Cancer	BC	BC or the other	ER-BC	HER2- hormone negative BC
Common	896	389	128	198 (BC or CC) 159 (BC or OC)	31 (↑9, ↓21)	31(↑9, ↓22)
TNBC	797	282	-	135 (BC or OC)	-	-
ER+HER2-BC	1403	460	-	223 (BC or CC) 172 (BC or OC)	-	26(↑6, ↓20)

Note: Breast Cancer (BC), Colorectal Cancer (CC), Ovarian Cancer (OC), ER negative (ER^-^), HER2 negative (HER2^-^).

We further examined the cancer-related genes in each category. Among the 896 genes in the common set, we observed 410 genes in Cancer, and 503 genes in Organismal Injury and Abnormalities. We found 389 genes in these two categories were associated with cancer; 128 genes were associated with BC; and 31 genes were associated with estrogen negative BC, 30 genes were associated with HER2^-^ hormone receptor negative BC and 31 genes were associated with HER2^-^BC. The unique genes significantly associated with Cell cycle, Cellular assembly and organization, DNA replication, recombination and repair are illustrated by a network in Fig 3A.

Among the 797 genes uniquely identified in TNBC, 294 and 381 genes are categorized in Cancer, and Organismal Injury and Abnormalities, respectively. We found 282 of these genes are associated with cancer and 135 genes are associated with breast cancer or colorectal cancer. The unique genes significantly associated with Cancer and Organismal Injury and Abnormalities are illustrated by a network in Fig 3B.

Among the 1403 genes uniquely identified by ER^+^HER2^-^BC, we found 498 genes in Cancer, 609 genes in Organismal Injury and Abnormalities, and 278 genes in Cellular growth and proliferation. We found 460 genes are associated with cancer; 223 genes are associated with BC or colorectal cancer, and 172 genes are associated with BC or ovarian cancer. More interestingly, we identified additional 26 genes besides 30 genes in common set that are also associated with HER2^-^ hormone receptor negative BC. These genes could be potential biomarkers for the diagnosis of ER^+^HER2^-^BC subtype. The unique genes significantly associated with Cancer and Organismal Injury and Abnormalities are illustrated by a network in Fig 3C.

### 7. Canonical pathways identified for TNBC and ER^+^HER2^-^BC

**Fig 4:**
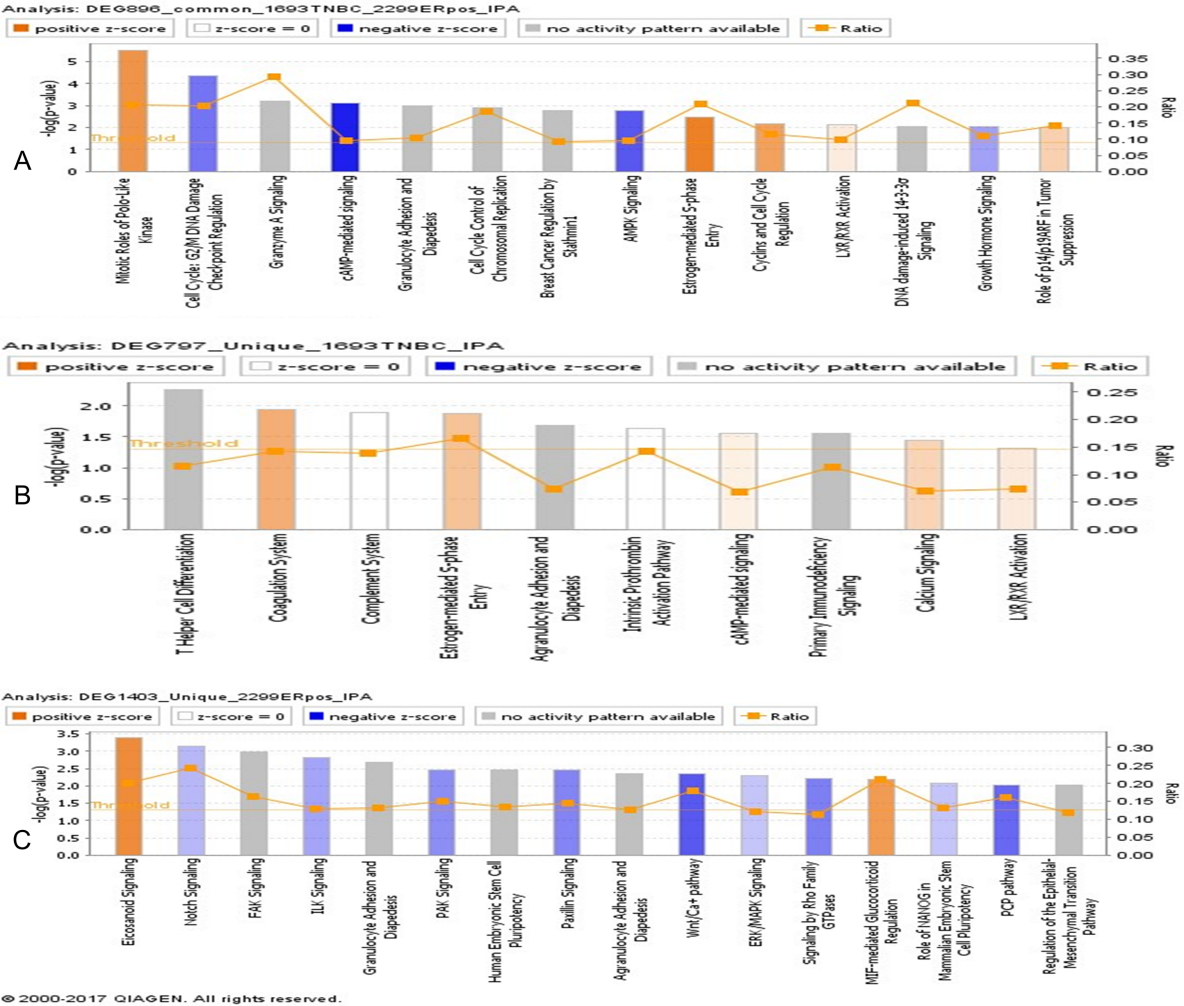
Canonical pathways identified by IPA. (A) Illustrated are the canonical pathways based on 896 DEGs commonly identified in TNBC and ER^+^HER2^-^BC. (B) Illustrated are the canonical pathways ions based on 797 DEGs uniquely identified in TNBC subtype. (C) Illustrated are the canonical pathways based on 1403 DEGs uniquely identified in ER+HER2-BC subtype.

We further examined the canonical pathways and biomarkers based on the three sets of DEGs from TNBC and ER^+^HER2^-^BC. For the common 896 DEGs, we identified the pathways highlighted by Cell Cycle with the DNA damage, cAMP-mediated intracellular signaling and Estrogen-mediated S-phase Entry (Fig 4A). For the 797 DEGs in TNBC, we identified four cancer-related pathways highlighted by cAMP-mediated Signaling, Calcium Signaling and LXR/RXR Activation (Fig 4B). These pathways play important roles in the regulation of cell cycle, promoting cell growth and proliferation or survival and apoptosis, and cell signaling. For example, cAMP-mediated intracellular signaling activates *ERK* via *EPAC1*, while *Src* and *Stat3* are activated by *Gai* and *Gao*. The persistent activation of these genes such as *Stat3* also mediates tumor-promoting inflammation. More interestingly, we identified several immuno-related signaling pathways including T Helper Cell Differentiation, Complement System, Agranulocyte Adhesion and Diapedesis and Intrinsic Prothrombin Activation Pathway (Fig 4B). These pathways play crucial roles in immune surveillance within the tumor microenvironment. Moreover, these pathways particularly T helper cell differentiation pathway may be related to current immunotherapeutic efficacy such as immunocheckpoint blockade inhibitors in TNBC.

For the 1403 DEGs in ER^+^HER2^-^BC, we identified many significant and cancer-related pathways that were not found in TNBC including Notch Signaling, *FAK* Signaling, *ILK* Signaling, *HER2*^*-*^ Signaling in Breast Cancer, *PAK* Signaling, *Paxillin* Signaling, and *Wnt/Ca*^+^ Signaling, *ERK*/*MAPK* Signaling, and *PCP* Signaling (Fig 4C). These signaling pathways are associated with cellular growth, proliferation and organismal development. For example, *Wnt/Ca*^+^ Signaling is involved in various aspects of cell development like cell differentiation, growth and proliferation. Again, we noted that a fewer number of the pathways associated with cellular immune response were identified including Granulocyte Adhesion and Diapedesis and Agranulocyte Adhesion and Diapedesis. Theses pathways may play a crucial role in helping ER^+^HER2^-^BC to grow and penetration via an inflammatory response as the report in the recent studies.

## Discussion

Using three independent BC datasets, our study reveals important considerations in the analysis of RNA-seq data. Because a type I error is usually considered to be a more serious error which one would like to avoid [35], it is important to control this error while maintaining a high sensitivity. Failing to do so can has a profound effect on the number of genes that are claimed as DEGs, resulting in a misleading biological interpretation. Using a nominal FDR at an acceptable level for controlling the type I error rate of α is the best approach for identifying DEGs in the analysis of RNA-seq. Current methods use an FDR of 0.05 to correct p-values in the presence of multiple genes. However, previous comparative analyses including the commonly used *DESeq* and *edegR* methods reported that these methods failed to maintain the actual FDR below the nominal value of 0.05, leading to an inflated type I error rate [23,36]. In a comparison of normalization methods (DESeq in *DESeq2*, TMM in *edgeR*, FQ, Med-pgQ2 and UQ-pgQ2) using MAQC2, we recently showed that Med-pgQ2 and UQ-pgQ2 performed best by achieving a smaller actual FDR and higher specificity while maintaining high sensitivity [28]. In addition, we also reported that *DESeq2* performed best in terms of achieving an actual FDR, specificity and sensitivity at a quantile cutoff of the mean read counts below the 75th percentile. These studies suggest that Med-pgQ2 or UQ-pgQ2 are relatively conservative for high gene read counts and *DESeq2* is relatively conservative for gene expression below the 75th percentile. Taking into consideration sensitivity and specificity, in our analysis scheme, we proposed a new and optimal approach to perform the DEG analysis of BC data. We utilized UQ-pgQ2 and *DESeq2* methods and robustly identify DEGs for GEO TNBC and ER^+^HER2^-^BC versus their controls. DEGs identified in this manner are deemed to be truly and differentially expressed.

Since true DEGs are unknown, and sensitivity rate is unable to be calculated, our study is mainly based on the discovery of false positives. Although this may be a limitation, we know from the previous study that the sensitivity rates for DEG analysis of MAQC2 from *DESeq2*, *edgeR* and UQ-pgQ2 methods were more than 90% given a 0.05 nominal FDR[28]. Based upon this and the previous studies comparing the existing methods for DEG analysis, we chose three methods (UQ-pgQ2, *DESeq2* and *edgeR*) to perform a within-group analysis. We demonstrated that UQ-pgQ2 normalization using a Wald statistical test from *DESeq2* performed best for the control of FP genes in the analysis of these BC dataset for any given |logFC| cutoff and a nominal FDR of 0.05. We found *DESeq2* is also a good method to analyze these BC datasets in terms of the number of DEGs detected with slightly higher false positives than UQ-pgQ2 method. As expected, these findings are consistent with previous studies. Furthermore, based on the FPR obtained from within group analysis, an optimal |logFC| cutoff was determined, which is further used to control FPR for the analysis.

Furthermore, Gene-expression profiling analysis has been used to dissect the heterogeneity of BC into six subtypes: Luminal A (ER^+^, low grade), Luminal B (ER^+^; high grade), HER2 positive (HER2^-^amplification), basal-like (ER^-^; HR^-^; HER2^-^), normal-like and most recent “claudin low” subtypes [37–40]. The results of our analysis of gene expression profiles for two BC subtypes (GEO TNBC and ER^+^HER2^-^BC) demonstrated that their gene signatures were significantly different. We identified 1,693 protein coding genes with a |logFC| ≥2 from the 42 TNBC patients compared to 21 paired control samples and 872 unique protein coding genes that were not identified in ER^+^HER2^-^BC. We also identified 2,299 protein coding genes with a |logFC| ≥1.5 from the 42 ER^+^HER2^-^BC patients compared to 30 paired control samples and 1042 protein coding genes uniquely expressed in ER^+^HER2^-^BC. With the aid of IPA, these DEGs were categorized in Cancer, and Organismal Injury and Abnormalities among the top diseases and biological functions. For the pathway analysis, we also identified unique pathways of each set that were associated with cancer cell growth, proliferation and development or were involved in cellular immune responses.

## Conclusions

Taken together, our combined approach with UQ-pgQ2 and *DESeq2* methods improves the performance on the analysis of the BC RNA-seq data with a control of false positives below the nominal level. With this approach, we have confidently identified two distinct gene expression patterns during the analysis of two BC subtypes (TNBC and ER^+^HER2^-^BC) downloaded from GEO. These cancer-related DEGs may serve as potential biomarkers for the diagnosis of BC, BC subtype or potential targets for the immunotherapy treatment in BC.

## Supporting information

S1 Table DEGs identified via within-group and between groups comparisons of TCGA-BRCA data from UQ-pgQ2, *DESeq2* and *edgeR*. This file is a word text file. DEGs are based on a different |log FC| cutoff given a nominal FDR≤0.05.
(DOCX)

S2 Table DEGs identified via within-group comparison of TCGA-TNBC subtype from UQ-pgQ2, *DESeq2* and *edgeR*. This file is a word text file. DEGs are based on a different |log FC| cutoff given a nominal FDR≤0.05.
(DOCX)

S3 Table DEGs commonly identified in TNBC and ER^+^HER2^-^BC. This file is a tab-delimited text file and contains 896 DEGs with FDR <0.05 and |logFC| > 1.5.
(XLS)

S4 Table DEGs uniquely identified in TNBC. This file is a tab-delimited text file and contains 797 DEGs with FDR <0.05 and |logFC| > 1.5.
(XLS)

S5 Table DEGs uniquely identified in ER^+^HER2^-^BC. This file is a tab-delimited text file and contains 1403 DEGs with FDR <0.05 and |logFC| > 1.5.
(XLS)

S1 Fig A Venn diagram illustrated the common and unique protein coding genes between 1693 DEGs in TNBC and 2299 DEGs in ER^+^HER2^-^BC. (TIF)
