# Supplementary material for: A combined approach with gene-wise normalization improves the analysis of RNA-seq data in human breast cancer subtypes
Source: PLoS One. 2018 Aug 8;13(8):e0201813. doi: 10.1371/journal.pone.0201813 (PMC6082555; doi:10.1371/journal.pone.0201813)
Supplement: S1 Table — This file is a word text file. DEGs are based on a different |log FC| cutoff given a nominal FDR≤0.05. (DOCX) [file pone.0201813.s001.docx]

**Table S1: DEG analysis performed via within-group and between-group comparisons for TCGA-BRCA data from three methods.** The DEGs are determined given a nominal FDR$\leq$0.05.

| Cutoff | Compared groups | UQ-pgQ2 | *DESeq2* | *edgeR* |
| --- | --- | --- | --- | --- |
| \|Log(FC)\|$\geq$1 | 58 BC vs. 59 BC | 70$\pm$97 | $120\pm$160 | 2,019$\pm$789 |
|  | 56ctr vs. 56 ctr | 2±3.3 | 4$\pm$5 | 513$\pm$47 |
|  | 117 BC vs. 112 ctr | 6,578 | 7,041 | 11,729 |
| \|Log(FC)\|$\geq$1.5 | 58 BC vs. 59 BC | 26$\pm$27 | 52$\pm$59 | 1,510$\pm$514 |
|  | 56ctr vs. 56 ctr | 1±1 | 2±2 | 400$\pm$31 |
|  | 117 BC vs. 112 ctr | 3,637 | 3,841 | 7,631 |
| \|Log(FC)\|$\geq$2 | 58 BC vs. 59 BC | 8$\pm$10 | 18$\pm$23 | 1,050$\pm$ 514 |
|  | 56ctr vs. 56 ctr | 0 | 0 | 308$\pm$ 21 |
|  | 117 BC vs. 112 ctr | 2,148 | 2,208 | 5,187 |
| \|Log(FC)\|$\geq$2.5 | 58 BC vs. 59 BC | 3$\pm$4 | 6$\pm$9 | 717$\pm$ 194 |
|  | 56ctr vs. 56 ctr | 0 | 0 | 249$\pm11$ |
|  | 117 BC vs. 112 ctr | 1,271 | 1,322 | 3,593 |
| \|Log(FC)\|$\geq$3 | 58 BC vs. 59 BC | 1$\pm$1 | 2$\pm$3 | 504$\pm$ 130 |
|  | 56ctr vs. 56 ctr | 0 | 0 | 206$\pm6$ |
|  | 117 BC vs. 112 ctr | 747 | 760 | 2,504 |
