# Supplementary material for: A combined approach with gene-wise normalization improves the analysis of RNA-seq data in human breast cancer subtypes
Source: PLoS One. 2018 Aug 8;13(8):e0201813. doi: 10.1371/journal.pone.0201813 (PMC6082555; doi:10.1371/journal.pone.0201813)
Supplement: S2 Table — This file is a word text file. DEGs are based on a different |log FC| cutoff given a nominal FDR≤0.05. (DOCX) [file pone.0201813.s002.docx]

**Table S2: FP gene analysis performed via within-group comparison of 122 TCGA-TNBC data from three methods.** The FP genes are obtained from 61TCGA-TNBC vs. 61 TCGA-TNBC given a nominal FDR$\leq$0.05.

| Cutoff | UQ-pgQ2 | *DESeq2* | *edgeR* |
| --- | --- | --- | --- |
| \|Log(FC)\|$\geq$1 | 93$\pm$90 | $143\pm$155 | 1,960$\pm$1375 |
| \|Log(FC)\|$\geq$2 | 56$\pm$38 | 90$\pm$82 | 1,635$\pm$ 1051 |
| \|Log(FC)\|$\geq$3 | 6$\pm$2 | 10$\pm$5 | 773$\pm$ 434 |
| \|Log(FC)\|$\geq$4 | 0 | 0 | 306$\pm$ 155 |
