# Supplementary figures and images for: A combined approach with gene-wise normalization improves the analysis of RNA-seq data in human breast cancer subtypes
Source: PLoS One. 2018 Aug 8;13(8):e0201813. doi: 10.1371/journal.pone.0201813 (PMC6082555; doi:10.1371/journal.pone.0201813)

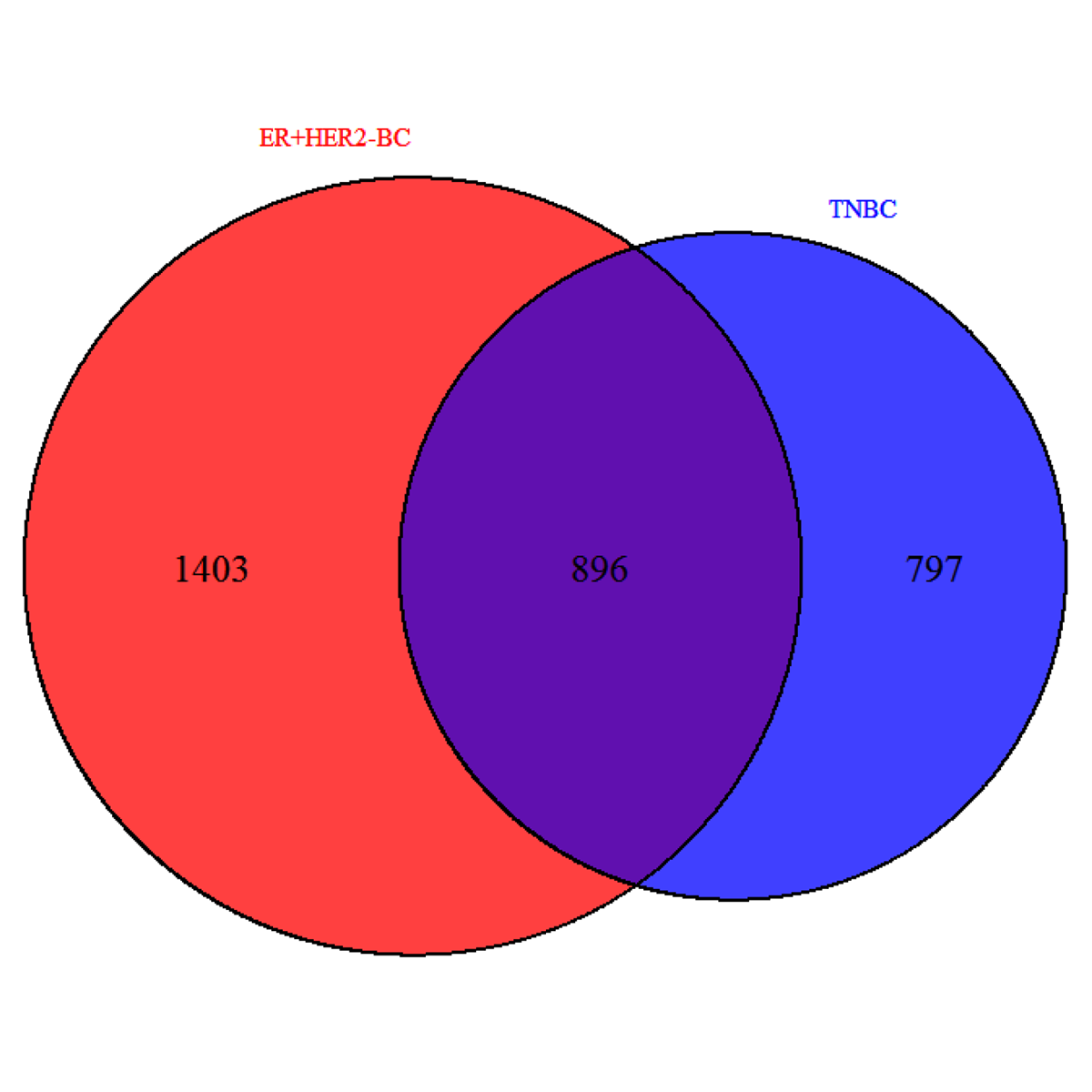

Supplement: S1 Fig — (TIF) [file pone.0201813.s006.tif]
